# Identification of a glycolysis‐related gene signature associated with clinical outcome for patients with lung squamous cell carcinoma

**DOI:** 10.1002/cam4.3945

**Published:** 2021-05-15

**Authors:** Ziming Xu, Shiwei Zhang, Fulai Nian, Shangyu Xu

**Affiliations:** ^1^ Department of Thoracic Surgery Wuxi 9th People’s Hospital affiliated to Soochow University Wuxi Jiangsu China; ^2^ Department of Neurosurgery The Second Affiliated Hospital and Yuying Children's Hospital of Wenzhou Medical University Wenzhou Zhejiang China

**Keywords:** gene signature, glycolysis, lung squamous cell carcinoma, survival

## Abstract

**Background:**

Lung squamous cell carcinoma (LUSC), one of the main types of lung cancer, has caused a huge social burden. There has been no significant progress in its therapy in recent years, Resulting in a poor prognosis. This study aims to develop a glycolysis‐related gene signature to predict patients’ survival with LUSC and explore new therapeutic targets.

**Methods:**

We obtained the mRNA expression and clinical information of 550 patients with LUSC from the Cancer Genome Atlas (TCGA) database. Glycolysis genes were identified by Gene Set Enrichment Analysis (GSEA). The glycolysis‐related gene signature was established using the Cox regression analysis.

**Results:**

We developed five glycolysis‐related genes signature (HKDC1, AGL, ALDH7A1, SLC16A3, and MIOX) to calculate each patient's risk score. According to the risk score, patients were divided into high‐ and low‐risk groups and exhibited significant differences in overall survival (OS) between the two groups. The ROC curves showed that the AUC was 0.707 for the training cohort and 0.651 for the validation cohort. Additionally, the risk score was confirmed as an independent risk factor for LUSC patients by Cox regression analysis.

**Conclusion:**

We built a gene signature to clarify the connection between glycolysis and LUSC. This model performs well in evaluating patients’ survival with LUSC and provides new biomarkers for targeted therapy.

## Graphical abstract

We established a glycolysis‐related gene signature to calculate the risk score for patients with lung squamous cell carcinoma. The risk score divided patients into high‐ and low‐risk groups, and there was a significant difference in survival between the two groups. In addition, the risk score was confirmed as an independent predictor of patients.

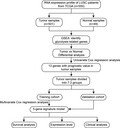

## 1 INTRODUCTION

Lung cancer has an exceptionally high incidence and mortality among all tumors worldwide. It is predicted that there would be 228,820 cases newly diagnosed with lung cancer, and 135,720 cases would die from this tumor in 2020, causing a tremendous social burden.^1^ Lung squamous cell carcinoma (LUSC) and lung adenocarcinoma constitute the central part of lung cancer and the former accounts for approximately 25%–30% of all lung. In recent years, the survival of patients with advanced lung adenocarcinoma has been dramatically improved due to molecular targeted therapy's progress. However, only a small proportion of LUSC molecules have been identified, leading to its current treatment plan limited to platinum‐based chemotherapy.^2^ The limited therapeutic effect causes a 5‐year survival rate lower than 5% for patients with LUSC. Hence, it is required further to clarify the underlying mechanism of the occurrence of LUSC and design new treatment strategies.

With the development of sequencing technology, many studies have confirmed that some molecular markers play a role in LUSC. These biomarkers were not only involved in transcription level but also a posttranscription level. For instance, circTP63 was reported to be upregulated in LUSC and associated with patients’ tumor stage and size. It attenuated the inhibition of FOXM1 by competitively binding to miR‐873‐3p, thus promoting tumor cell cycle progression.^3^ Another study highlighted the critical role of miRNA in LUSC. They found six genes and 12 miRNAs closely associated with the OS of patients with LUSC, which may be considered new therapeutic targets.^4^

Additionally, lncRNA HULC was confirmed to promote LUSC by regulating the PTPRO/NF‐κB pathway.^5^ These studies have deepened our understanding of the pathogenesis of LUSC and guided prognosis and targeted therapy. However, a single biomarker's efficacy in predicting the prognosis of patients with LUSC may be insufficient. Some researchers suggested that the establishment of multigene signatures may be a suitable option to evaluate cancer patients’ prognosis.^6^

One of the “hallmarks of cancer” is disrupted energy metabolism, characterized by mainly relying on glycolysis.^7^ The “Warburg effect” revealed that the rapid growth of tumor cells depended on efficient glycolysis to produce energy.^8^ A large number of studies have found the association between glycolysis and various tumors. Altenberg et al. ^9^ found that glycolysis genes were ubiquitously overexpressed in 24 types of tumors. Eight out of ten glycolytic enzymes were upregulated in lung cancer. Another study^10^ proposed a nine glycolysis‐related genes signature to evaluate the metastasis and prognosis for lung adenocarcinoma. It is convincing that glycolysis‐related genes may be a potential mechanism in the occurrence of lung cancer. But there is currently a lack of study linking LUSC with glycolysis genes.

In this study, we used the expression profile of 550 patients with LUSC obtained from TCGA. GSEA was used to find glycolysis genes, and a 5‐gene signature was constructed. We further tested the performance of the gene signature in the training and validation set, respectively. These findings reveal a close association between LUSC and glycolysis and demonstrate the possibility of using glycolysis‐related gene signatures to assess patients’ prognosis with LUSC.

## 2 METHODS

### 2.1 Data sources

**FIGURE 1:**
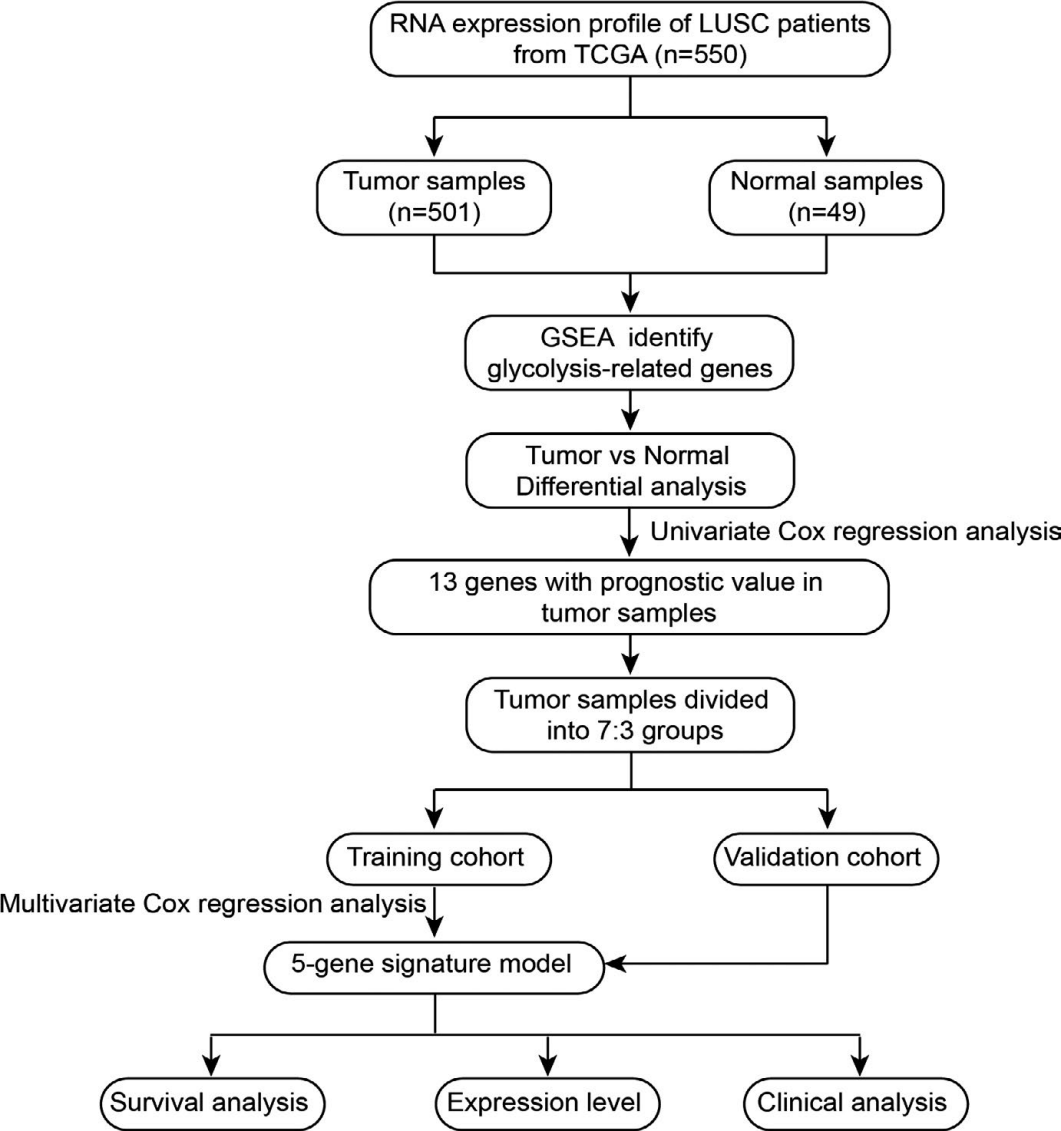
The flowchart of this study

The overall design of this study was exhibited in the flowchart (Figure 1). Data of 501 patients with LUSC and 49 normal samples, including RNA‐sequencing data (FPKM value) and clinical features, were extracted from the Genomic Data Commons (https://portal.gdc.cancer.gov/), which linked to The Cancer Genome Atlas (TCGA) database. The clinical features recorded were mainly age, gender, tumor stage, and TNM classification. Also, each case is accompanied by a detailed follow‐up time and overall survival. Table 1 describes the detailed clinical information of all patients.

**TABLE 1:** Clinical features of patients with lung squamous cell carcinoma

Characteristic	Train group	Test group	*p* value
Age (years)			0.884
≤65	131	56	
>65	211	87	
Unknown	4	1	
Gender			0.765
Male	255	108	
Female	91	36	
AJCC stage			0.410
I	170	70	
II	114	42	
III	54	29	
IV	4	3	
Unknown	4	0	
T stage			0.471
T1	78	34	
T2	205	81	
T3	50	19	
T4	13	10	
N stage			0.345
N0	220	94	
N1‐3	121	50	
Unknown	5	0	
M stage			0.571
M0	282	120	
M1	4	3	
Unknown	60	21	

### 2.2 Gene set enrichment analysis (GSEA)

GSEA software 4.0.3 was used to define significantly different glycolysis‐related gene sets between patients with LUSC and normal samples. We downloaded six gene sets related to glycolysis from the Molecular Signatures Database (https://www.gsea‐msigdb.org/gsea/msigdb/index.jsp). Each gene set had 1000 permutations to get a normalized enrichment score (NES). Normalized *p*‐value <0.05, FDR <0.1, and NES >1.6 were used to determine whether the gene set was selected for subsequent analysis. We integrated gene sets with significant differences and finally got 311 genes related to glycolysis.

### 2.3 Development and validation of the glycolysis‐related gene signature

We performed a differential analysis of these 311 genes in normal samples and patients with LUSC by the Mann–Whitney–Wilcoxon test (*p* < 0.05). Univariate Cox regression analysis was used to identify genes significantly related to OS, and genes with *p* < 0.05 were selected for subsequent multivariate Cox regression analysis. We randomly divided the tumor samples into 7:3 groups and used them as the training set and the validation set, respectively. We performed a multivariate Cox regression analysis in the training cohort and built a five glycolysis‐related genes signature. Next, a linear combination of the expression of five genes weighted with the regression coefficient formed a risk scoring system. The formula was as follows:

$\begin{array}{cc} \text{Risk score} & =\text{expression of the gene}\;1\;\times \;\beta 1\\ & \;+\text{expression of the gene}\;2\;\times \;\beta 2+\;\ldots \\ & \;+\;\text{expression of the gene}\;n\;\times \;\beta n \end{array}$ β2 βn.

Patients with LUSC in the training cohort were divided into high‐ or low‐risk groups based on the median risk score. The 5‐gene signature and the median risk score were also used in the validation cohort.

### 2.4 GO and KEGG pathway enrichment analyses

To investigate the specific function of differentially expressed genes (DEGs), we conducted Gene Ontology (GO) and Kyoto Encyclopedia of Genes and Genomes (KEGG) enrichment analyses. We visualized the top 20 significant terms for GO analysis and top 10 significant terms for KEGG analysis, respectively, using the clusterProfiler R packages. We identified the terms based on the cutoff of *p*‐value < 0.01 and Benjamin–Hochberg adjusted *p*‐value < 0.05 as significant terms.

### 2.5 Immunohistochemistry and lung cancer cell lines

Immunohistochemistry staining results were extracted from the Human Protein Atlas (https://www.proteinatlas.org/). The expression of glycolysis‐related genes were compared in lung squamous cell carcinoma samples and normal lung tissue. The expression data of glycolysis‐related genes in cell lines were obtained from the Cancer Cell Line Encyclopedia (CCLE) database (https://portals.broadinstitute.org/ccle). It mainly included 136 non‐small‐cell lung cancer cell lines and 54 small‐cell lung cancer cell lines.

### 2.6 Statistics

The chi‐square test was used for categorical variables. Kaplan–Meier curves and log‐rank tests were used to compare survival differences between the two groups. ROC curves were used to access the effectiveness of the gene signature in predicting OS. Independent prognostic factors were identified by univariate and multivariate Cox regression analyses. CBioPortal (http://www.cbioportal.org/) provided genetic variation of five glycolysis‐related genes. All statistical analyses and figures were mainly done by SPSS 24.0 and R 4.0.2 software. *p* < 0.05 was considered to be significant.

## 3 RESULTS

### 3.1 Initial screening of glycolysis‐related genes

**FIGURE 2:**
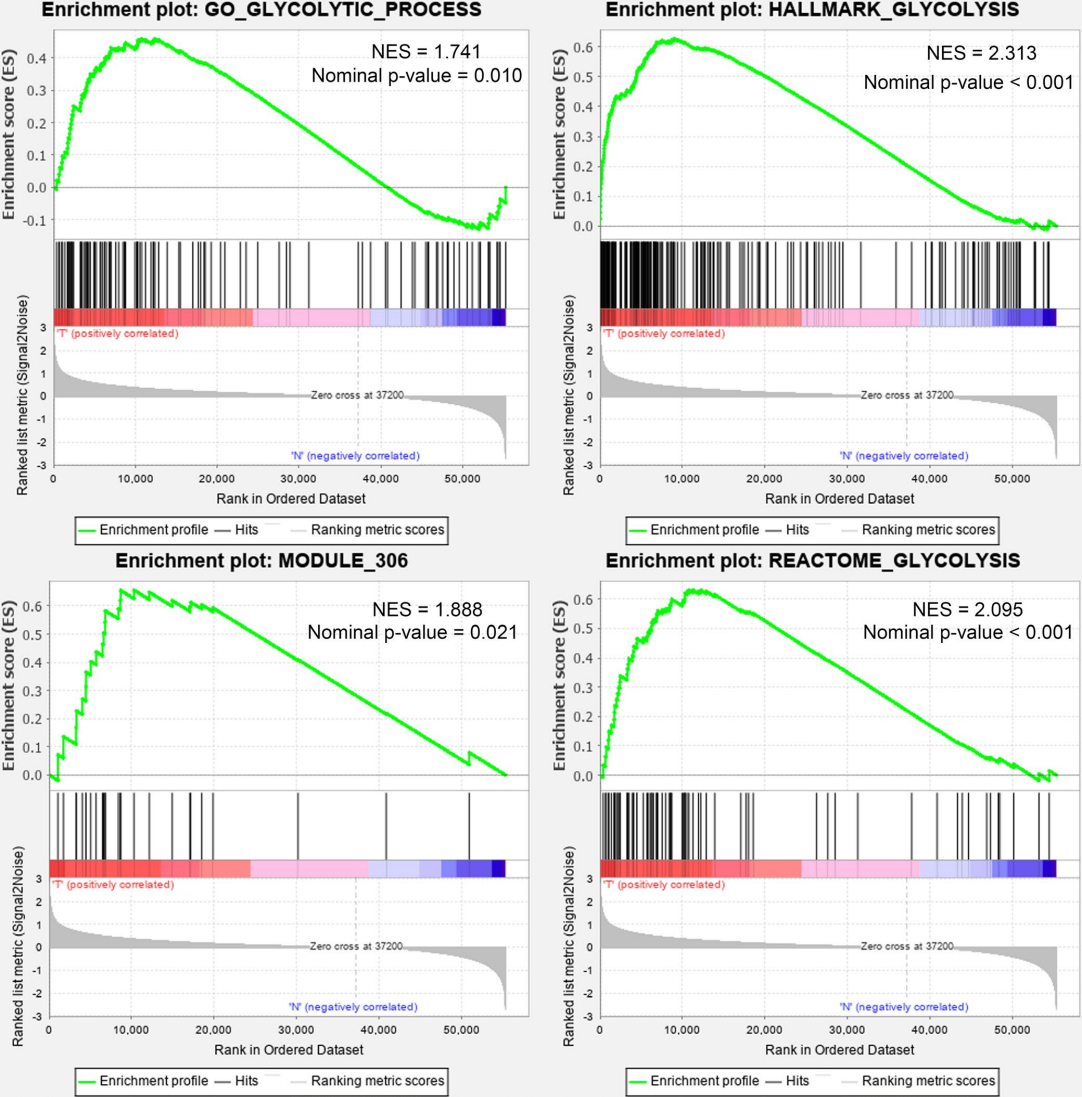
Enrichment plots of four glycolysis‐related gene set that were significantly differentiated between LUSC patients and normal samples. The GO_GLYCOLYTIC_PROCESS gene set had an NES of 1.741 and a *p* value = 0.010; the HALLMARK_GLYCOLYSIS gene set had an NES of 2.313 and a *p* value < 0.001; the MODULE_306 gene set had an NES of 1.888 and a *p* value = 0.021; the REACTOME_GLYCOLYSIS gene set had an NES of 2.095 and a *p* value < 0.001. Gene set data were from the Molecular Signatures Database (https://www.gsea‐msigdb.org/gsea/msigdb/index.jsp). NES: normalized enrichment score

Six glycolysis‐related gene sets were analyzed by the GSEA method to detect whether there were significant differences between samples with LUSC and normal samples. Four gene sets were enriched in samples with LUSC in our study. (Figure 2, GO_GLYCOLYTIC_PROCESS: NES = 1.741, nominal *p* = 0.010; HALLMARK_GLYCOLYSIS: NES = 2.313, nominal *p* < 0.001; MODULE_306: NES = 1.888, nominal *p* = 0.021; REACTOME_GLYCOLYSIS: NES = 2.095, nominal *p* < 0.001) We integrated all the genes in these four gene sets and obtained 311 glycolysis‐related genes for subsequent analysis.

### 3.2 Identification of glycolysis‐related genes relevant to OS of LUSC patients

**FIGURE 3:**
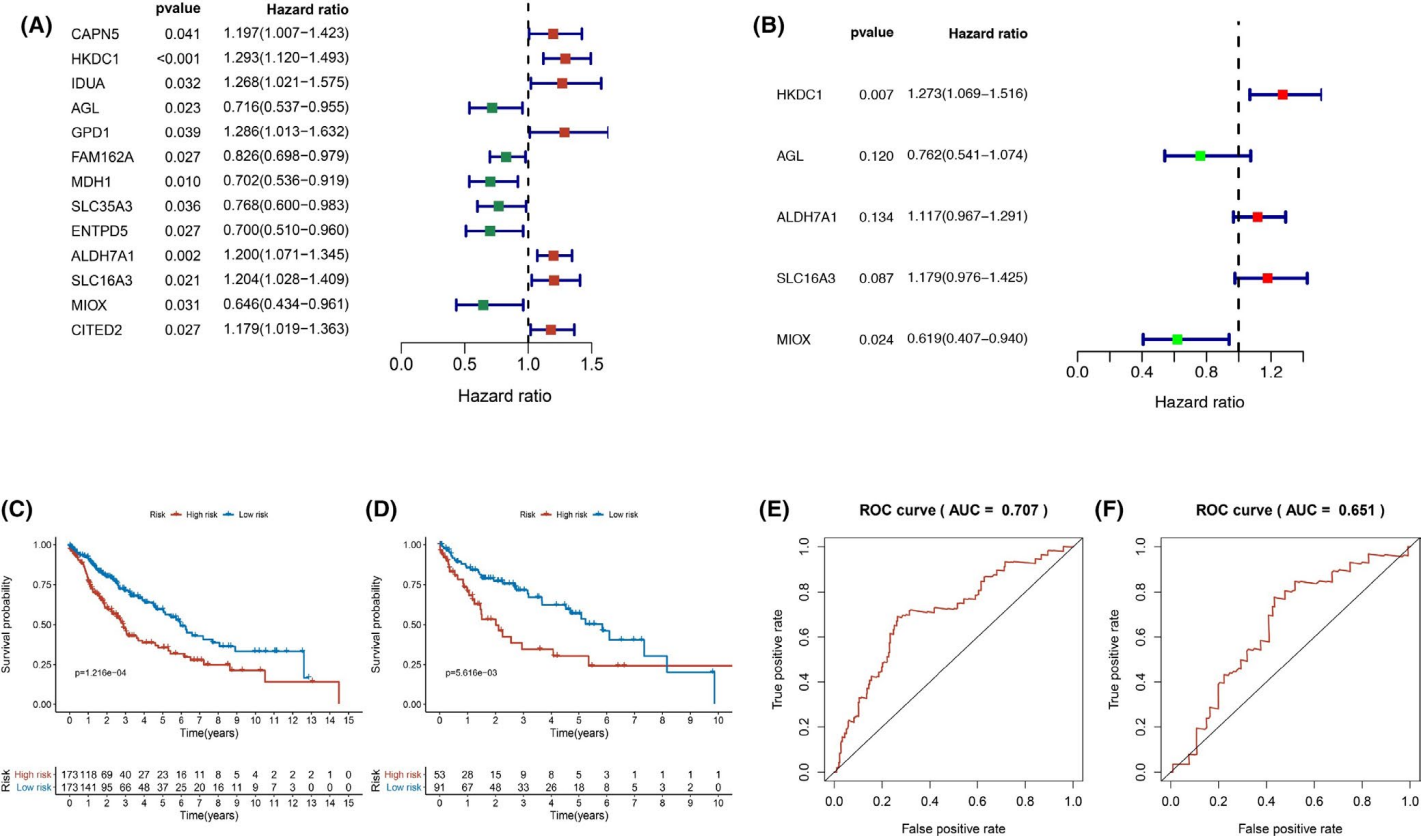
A, 13 glycolysis‐related genes were significantly related to prognosis in patients with LUSC by univariate Cox regression analysis. B, Multivariate Cox regression analysis showed that HKCD1 and MIOX were independent risk factor in the glycolysis‐related gene signature. C, Kaplan–Meier curves showed that high‐risk patients had poorer survival in the training cohort (*p* < 0.001). D, Kaplan–Meier curves showed high‐risk patients had worse survival in the validation cohort (*p* = 0.006). E, The AUC of the ROC curve was 0.707 in the training cohort. F, The AUC of the ROC curve was 0.651 in the validation cohort. ROC: Receiver Operating Characteristic. AUC: Area Under Curve

We first conducted a differential analysis of these 311 genes. There were 265 genes differentially expressed in samples with LUSC and normal samples (*p* < 0.05). Next, univariate Cox regression analysis was performed to acquire DEG, among which 13 genes were relevant to OS of patients with LUSC (Figure 3A). The association between these 13 genes and patients’ survival was further validated via multivariate Cox regression analysis in the training cohort. We found that HKDC1 (HR: 1.273, 95% CI: 1.069–1.516) and MIOX (HR: 0.619, 95% CI: 0.407–0.940) were confirmed as independent predictors for patients with LUSC (Figure 3B). Additionally, three filtered mRNA (HKDC1, ALDH7A1, and SLC16A3) appeared as risk factors with HR >1, whereas the other two filtered mRNA (AGL and MIOX) emerged as protective factors with HR <1 (Table 2).

**TABLE 2:** The information of five glycolysis‐related genes in the gene signature for patients with lung squamous cell carcinoma

Gene	Ensemble ID	Location	β (cox)	HR	*p* value
HKDC1	ENSG00000156510	chr10:70,980,059–71,027,315	0.241	1.273	0.007
AGL	ENSG00000162688	chr1:100,315,640–100,389,579	−0.272	0.762	0.120
ALDH7A1	ENSG00000164904	chr5:125,877,533–125,931,110	0.111	1.117	0.134
SLC16A3	ENSG00000141526	chr17:80,186,273–80,219,005	0.165	1.179	0.087
MIOX	ENSG00000100253	chr22:50,925,213–50,929,077	−0.480	0.619	0.024

### 3.3 GO and KEGG enrichment analyses of DEGs

**FIGURE 4:**
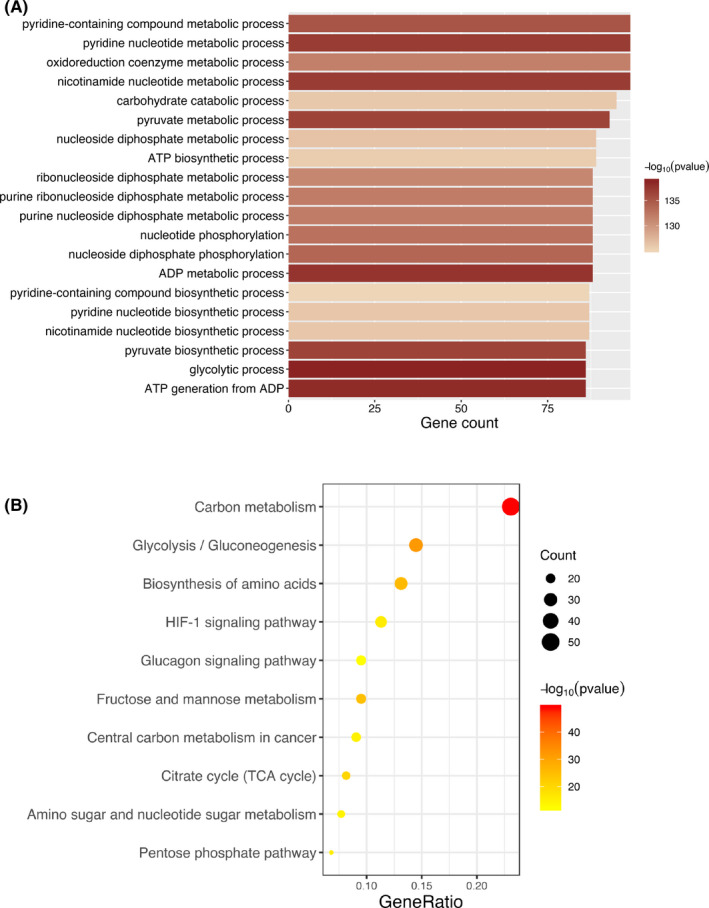
A, Top 20 GO‐BP terms. B, Top 10 KEGG terms. The length of the barplots and the size of the balls represented the number of genes enriched. The color depth represented the *p* value

To investigate the biological function of DEGs, we carried out GO and KEGG enrichment analyses exhibited the top 20 terms of GO and the top 10 terms of KEGG enrichment results. We found that the top GO‐BP terms (Figure 4A) were associated with energy metabolism, such as “ATP biosynthetic process” (gene count = 89, *p* = 1.69E−126), “glycolytic process” (gene count = 86, *p* = 1.19E−139), and “carbohydrate catabolic process” (gene count = 95, *p* = 8.77E−127). The results of KEGG enrichment analysis (Figure 4B) were mainly about metabolic reprogramming of tumor which were similar to that of GO‐BP, such as “citrate cycle (TCA cycle)” (gene count = 18, *p* = 2.37E−21), and some pathway which was reported to play important role in cancer progression, such as “carbon metabolism” (gene count = 51, *p* = 8.62E−50), “HIF‐1 signaling pathway” (gene count = 25, *p* = 8.10E−17), and “biosynthesis of amino acids” (gene count = 29, *p* = 1.26E−26).

### 3.4 Development of the 5‐genes signature to evaluate OS of the patient with LUSC

A predictive risk scoring system was built to calculate each patient's risk score based on the previous steps’ results. The formula was as follows:

Risk score = (expression of HKDC1 × 0.241) + (expression of AGL × −0.272) + (expression of ALDH7A1 × 0.111) + (expression of SLC16A3 × 0.165) + (expression of MIOS × −0.480). There was only one risk score for each patient with LUSC. We divided patients into high‐ and low‐risk groups based on the training Cohort's median risk score. Kaplan–Meier curves showed that high‐risk patients had poor OS than low‐risk patients in the training cohort (*p* < 0.001) (Figure 3C). Survival analysis of the validation cohort also showed similar results (*p* = 0.006) (Figure 3D).

**FIGURE 5:**
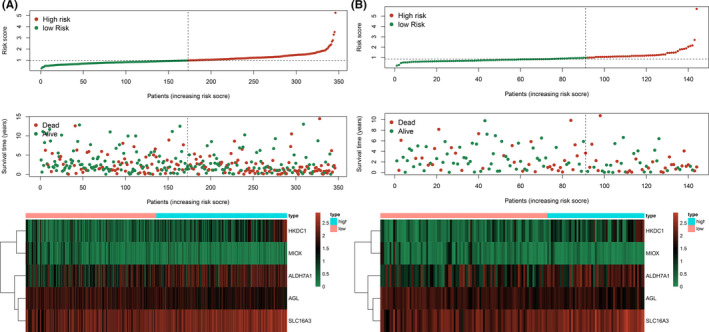
Risk score distribution, survival time, and heatmap of 5 genes’ expression profile for each LUSC patient. A, High‐risk patients had higher mortality and shorter survival time in the training cohort. HKDC1, ALDH7A1, and SLC16A3 were high expressed in high‐risk patients, whereas AGL and MIOX were low expressed in high‐risk patients; B, Similar results were observed in the validation cohort

Furthermore, the ROC curves exhibited that the AUC was 0.707 for the training set (Figure 3E) and 0.651 for the validation set, respectively (Figure 3F), which indicated good sensitivity and specificity of the 5‐genes signature in predicting OS for patients with LUSC. The distribution of risk score and survival information for each patient in the training cohort was shown in Figure 5 (Figure 5A for the training cohort and Figure 5B for the validation cohort), revealing that high‐risk patients had higher mortality and shorter survival time. The heatmap, respectively, displayed the differences in the expression profiles of the five genes between high‐risk and low‐risk groups in the training cohort and the validation cohort (Figure 5). As the risk score of patients with LUSC increased, the expression levels of risk genes (HKDC1, ALDH7A1, and SLC16A3) were obviously upregulated. In contrast, the expression levels of protective genes (AGL and MIOX) were downregulated.

### 3.5 A risk score based on the 5‐gene signature as an independent prognostic factor for LUSC patients

**FIGURE 6:**
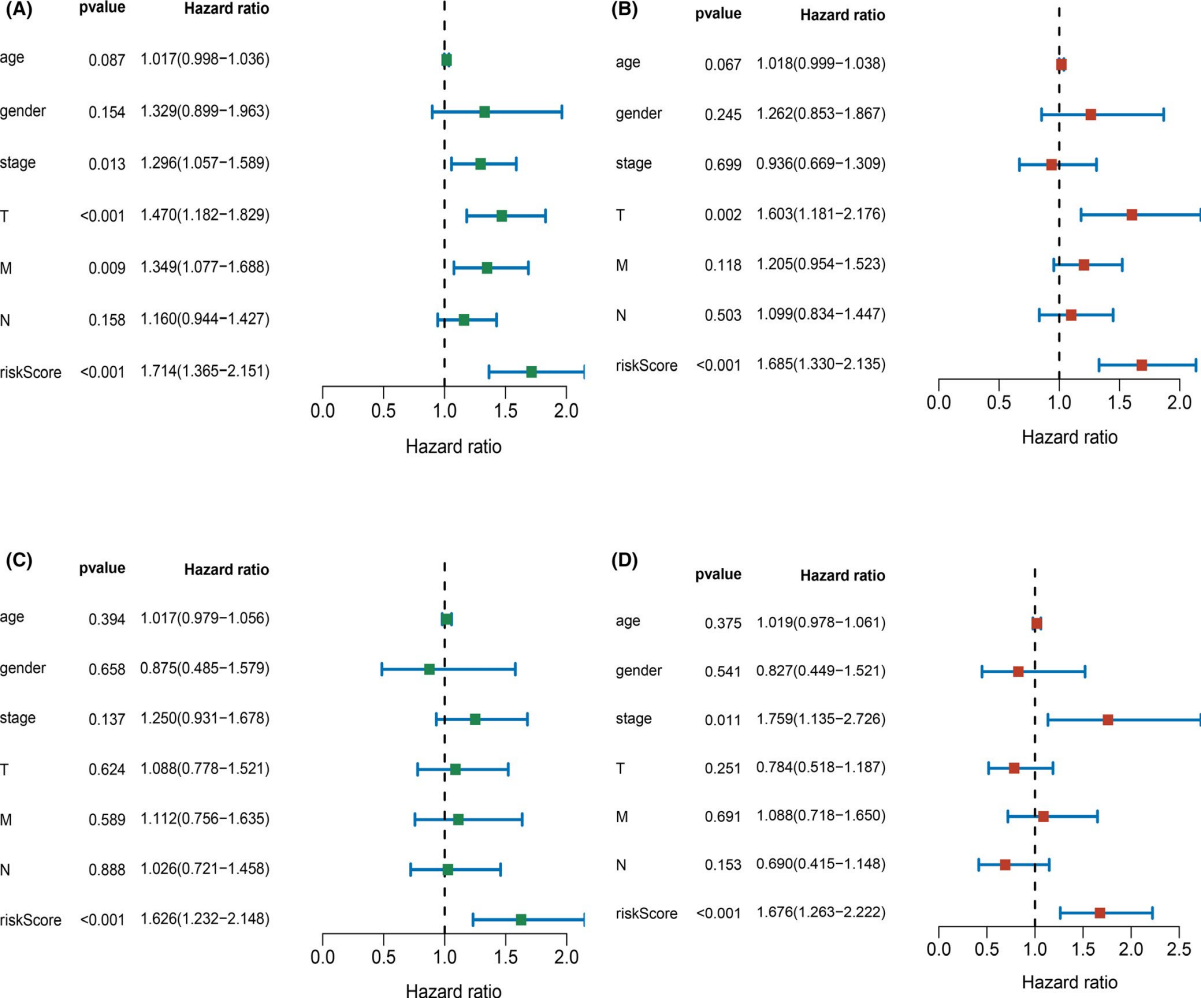
Univariable and multivariable Cox regression analyses for each clinical feature and risk score. A, Stage, T staging, M staging, and riskScore were significant prognostic factors for LUSC patients by univariable analysis in the training cohort; B, T staging and riskScore were significant prognostic factors for LUSC patients by multivariable analysis in the training cohort; C, Only riskScore was significant prognostic factor for LUSC patients by univariable analysis in the validation cohort; D, Stage and riskScore were significant prognostic factors for LUSC patients by multivariable analysis in the validation cohort

To evaluate the predictive value of risk scores derived from glycolysis‐related gene signatures, we used univariate and multivariate Cox regression analyses to identify whether the risk score and clinicopathological features were independent risk factors for OS of patients with LUSC. The clinical features of interest mainly included age, gender, American Joint Committee on Cancer (AJCC) stage, T stage, N stage, and M stage. The result of univariate Cox regression analysis of the training cohort revealed that AJCC stage (HR = 1.296, 95% CI: 1.057–1.589), T stage (HR = 1.470, 95%CI: 1.182–1.829), M stage (HR = 1.349, 95%CI: 1.077–1.688), and the risk score (HR = 1.714, 95%CI: 1.365–2.151) were associated with significantly low OS (Figure 6A), whereas multivariate Cox regression analysis showed that only T stage (HR = 1.603, 95%CI: 1.181–2.176) and the risk score (HR = 1.685, 95%CI: 1.330–2.135) were statistically significant (Figure 6B). For the validation cohort, univariate and multivariate Cox regression analyses showed that the risk score (univariate: HR = 1.626, 95%CI: 1.232–2.148; multivariate: HR = 1.676, 95%CI: 1.263–2.222) was correlated with worse OS of patients with LUSC (Figure 6C,D). These results indicated that the five glycolysis‐related gene signature was an independent prognostic factor for patients with LUSC.

### 3.6 Genetic alterations and expression levels of the five glycolysis‐related genes in LUSC

**FIGURE 7:**
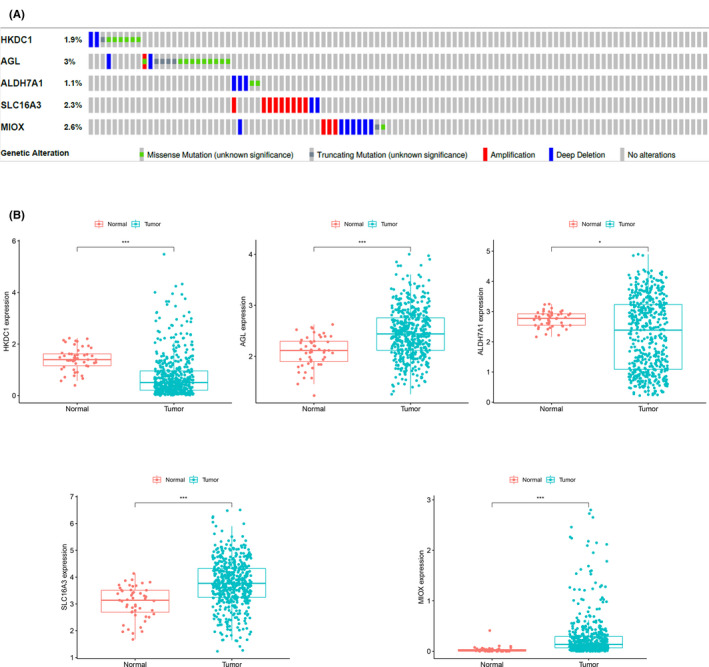
Selected genes‐specific alteration frequency (A) (data from CBioPortal: http://www.cbioportal.org/) and expression level between tumor samples and normal samples (B). AGL, SLC16A3, and MIOX were significantly high expressed in tumor samples, whereas HKDC1 and ALDH7A1 were significantly low expressed in tumor samples (data from the Genomic Data Commons: https://portal.gdc.cancer.gov/)

The cBioPortal database covers the genomic data of 487 patients with LUSC, providing genetic alterations in different genes. Among the 487 patients with LUSC, 7 had mutations and 2 had deep deletions in HKDC1; 14 had mutations, 1 had amplifications, and 2 had deep deletions in AGL; 2 had mutations and 3 had deep deletions in ALDH7A1; 9 had amplifications and 2 had deep deletions in SLC16A3; and 2 had mutations, 3 had amplifications, and 7 had deep deletions in MIOX (Figure 7A). The expression levels of the five glycolysis‐related genes in samples with LUSC and normal samples were also analyzed. The results showed that AGL, SLC16A3, and MIOX were significantly high expressed in tumor samples, whereas HKDC1 and ALDH7A1 were significantly low expressed in tumor samples (Figure 7B).

### 3.7 Subgroup analysis

**FIGURE 8:**
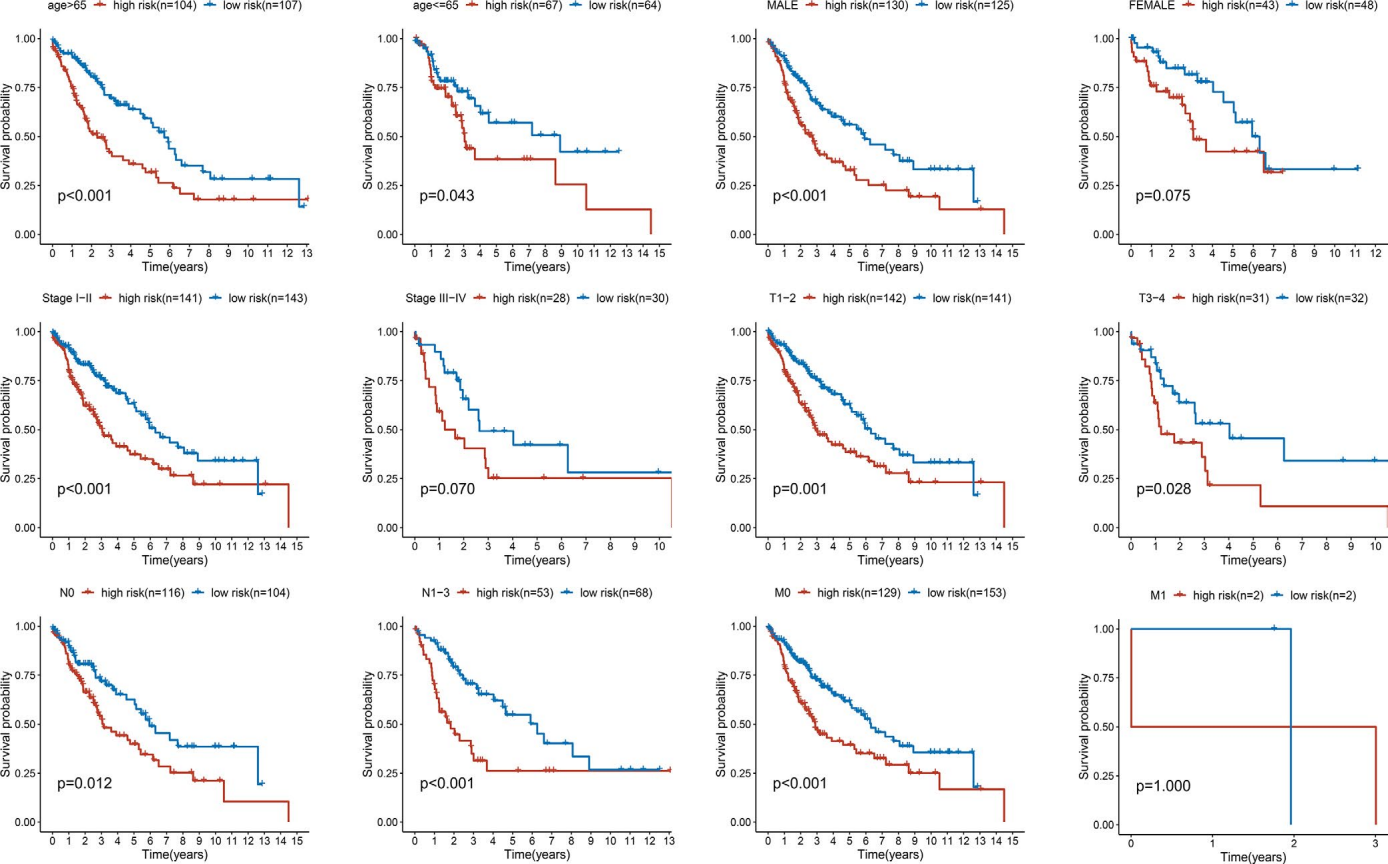
Kaplan–Meier curves for the predictive value of the risk score for LUSC patients divided by each clinical feature. High‐risk patients had a significantly worse survival than low‐risk patients in most subgroups except for the female (*p* = 0.075), stage III–IV (*p* = 0.070), and M1 subgroups (*p* = 1.000)

Next, we divided patients into different subgroups according to clinical features, such as age (>65 vs. ≤65 years), gender (male vs. female), AJCC stage (stage I–II vs. stage III–IV), T stage (T1–2 vs. T3–4), N stage (N0 vs. N1–3), and M stage (M0 vs. M1). The results showed that high‐risk patients had a significantly worse survival than low‐risk patients in most subgroups except for the female, stage III–IV, and M1 subgroups (Figure 8). It revealed that the five glycolysis‐related gene signature was suitable for multiple categories of patients with LUSC.

### 3.8 Validation of the glycolysis‐related genes in tissue samples and lung cancer cell lines

**FIGURE 9:**
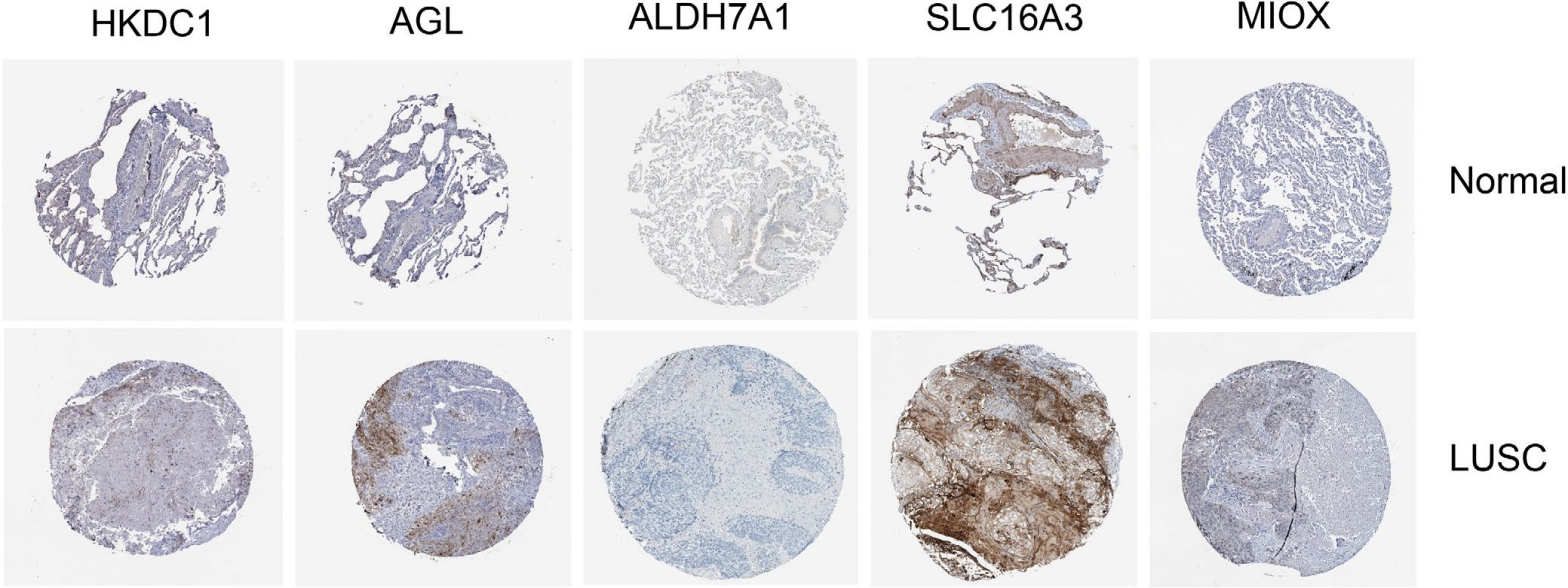
Validation of the gene signature by immunohistochemistry between LUSC and normal samples. AGL and SLC16A3 were significantly overexpressed in tumor samples compared to normal samples (data from the Human Protein Atlas: https://www.proteinatlas.org/)

**FIGURE 10:**
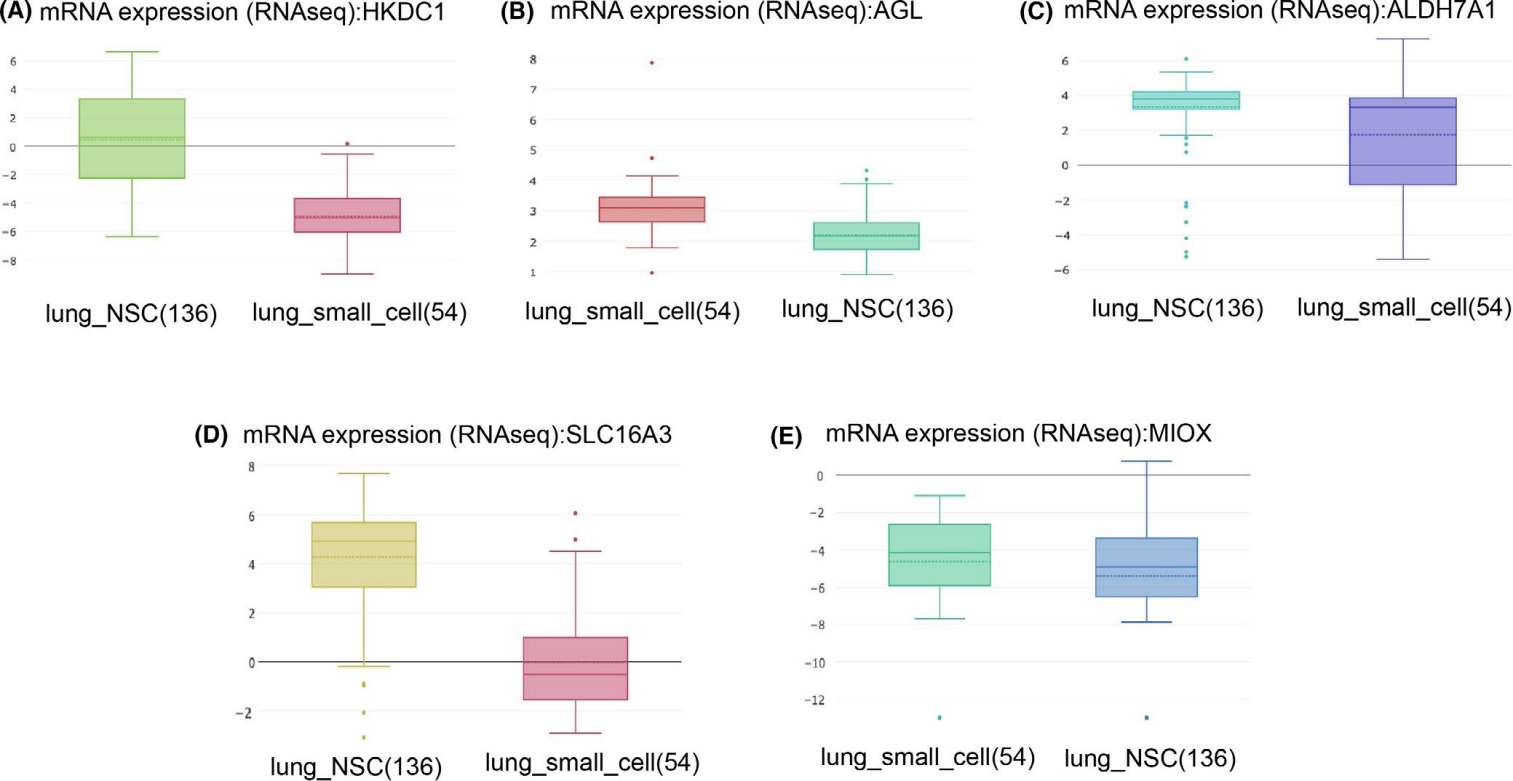
Validation of the gene signature by lung cancer cell lines between non‐small‐cell lung cancer cell line and small‐cell lung cancer cell line. HKCD1 and SLC16A3 were significantly overexpressed in non‐small‐cell lung cancer cell lines than small‐cell lung cancer cell lines (data from the Cancer Cell Line Encyclopedia database https://portals.broadinstitute.org/ccle)

To confirm the gene signature's reliability, we used immunohistochemical data to detect protein levels in five gene in normal lung tissue samples and lung cancer cell lines. The results showed that AGL and SLC16A3 were significantly overexpressed in tumor samples compared to normal samples (Figure 9). HKCD1 and SLC16A3 were significantly overexpressed in non‐small‐cell lung cancer cell lines than small‐cell lung cancer cell lines (Figure 10).

## 4 DISCUSSION

Previous studies had confirmed that some clinical characteristics, such as age, histological type, tumor size, tumor stage, and treatment, played a pivotal role in the prognosis of patients with LUSC and constructed predictive models.^11^, ^12^ With the popularization of genomics, researchers paid more attention to the impact of differences at the molecular level on patients’ prognosis with LUSC. They identified many molecular markers that were believed to be at the core of most cases of LUSC, involving protein, mRNA, lncRNA, circRNA, miRNA, and DNA methylation, etc. However, these studies often focused on the impact of a single molecule on LUSC, which were insufficient for monitoring the prognosis of patients with LUSC because the molecule was involved in multiple pathways and regulatory processes. A reasonable solution is to establish a gene signature, combining various genes’ expression to construct a prediction model. Several gene signatures for LUSC have also been proposed. For example, Li et al. ^6^ developed four methylation‐driven genes signature (GCSAM, GPR75, NHLRC1, and TRIM58) through multivariate Cox regression analysis and verified it in external data. Another study identified seven IncRNAs as potential prognostic factors for LUSC and constructed a prognostic signature using five of them (AC022148.1, HCG9, LINC00460, C5orf17, and LINC00261).^13^ In this study, we focused on glycolysis‐related genes, which had a crucial role in tumors.

In our study, GSEA analysis was carried out based on the mRNA data of 550 LUSC patients. We showed four glycolysis gene sets with significant differences (*p* < 0.05) between tumor samples and normal samples. We extracted the genes in these four glycolysis gene sets for subsequent analysis. Then, a 5‐gene signature was built to evaluate each patient's risk score. Subsequent verification confirmed that the risk score derived from the five glycolysis‐related genes signature could be used to classify patients’ risk with LUSC and predict patients’ prognosis.

As for the five glycolysis‐related genes we identified, HKDC1, a putative fifth hexokinase, was one of the rate‐limiting enzymes regulating glucose metabolism in several organisms.^14^ There was growing evidence indicating the association between HKDC1 and cancer susceptibility. Furthermore, it was plausible that HKDC1 could be a promising potential therapeutic target for numerous kinds of carcinomas. Li et al.^15^ reported that high levels of HKDC1 was a risk factor for patients with lung squamous cell carcinoma who tended to exhibit a worse prognosis. Chen et al.^16^ found that HKDC1 could promote breast tumor growth and transfer through the PGC1β/SREBP1 pathway.

ALDH7A1, an aldehyde dehydrogenase, functioned in the detoxification of aldehydes via lipid peroxidation and alcohol metabolism. Previous studies have identified the relationship between its role and the occurrence of non‐small‐cell lung carcinoma (NSCLC). ALDH7A1 was abundant in cancer stem cells, and knockdown of ALDH7A1 enhanced NSCLC sensitivity to cisplatin. What is more, Giacalone et al.^17^ found that high expression of ALDH7A1 led to a high recurrence rate in surgically treated patients.

SLC16A3, also known as MCT4, one member of solute carriers transporting monocarboxylate molecules, was reported to regulate tumor cell migration, invasion, and proliferation in numerous kinds of carcinomas.^18^, ^19^, ^20^ Moreover, SLC16A3 was regarded as a critical regulator for lactate metabolism in NSCLC cells based on aerobic glycolysis. Thus, SLC16A3 was believed to be a treatment site for glycolysis‐preference cancer cells.^21^

AGL was primarily responsible for breaking down glycogen and was suggested to be closely connected with bladder cancer. AGL was regarded as a biomarker that suppressed tumor growth in bladder cancer.^22^ AGL silencing promoted bladder tumor cell growth via different mechanisms such as promoting the synthesis of glycine^22^ and HAS2‐mediated hyaluronic acid (HA) synthesis.^23^ Furthermore, a recent study reported the function of AGL in NSCLC and suggested that the silencing of AGL enhanced NSCLC cells’ growth, which was mediated by HAS2.^24^

Myo‐inositol oxygenase (MIOX) was one member of the family consisting of different Aldo‐Keto reductases and participated in starting the myo‐inositol metabolism. Its overexpression was reported to induce ROS production.^25^, ^26^ The imbalance between the production and clearance of ROS caused oxidative stress, which ultimately led to tumorigenesis.^27^ Thus, although there was no study identifying the association between MIOX and cancer susceptibility, it was plausible that MIOX contributed to tumor development. The above studies revealed that these five glycolysis‐related genes are closely related to tumors, even lung cancer, supporting our gene signature's reliability for providing clues in the prognosis of patients with LUSC.

Although many biomarkers have been confirmed to be strongly associated with the occurrence and progression of LUSC, no signatures composed of the glycolysis‐related gene have been built yet. This study reports a gene signature for the first time based on glycolysis genes and is used for predicting the prognosis of patients with LUSC. Its predictive performance applies to patients with LUSC of various classifications and has good stability.

## 5 CONCLUSION

We developed a five glycolysis‐related genes signature (HKDC1, AGL, ALDH7A1, SLC16A3, and MIOX) to predict patients’ prognosis with LUSC. And we used internal data to verify its feasibility. The risk score derived from this gene signature was an independent predictor of patients with LUSC. We hope this signature can be used for clinical work and develop a new target treatment.

## CONFLICT OF INTEREST

The authors have no conflict of interest to declare.

## ETHICS APPROVAL STATEMENT

TCGA database belongs to public databases. The patients involved in the database have obtained ethical approval. Users can download relevant data for free for research and publish relevant articles. Our study is based on open‐source data, so there are no ethical issues and other conflicts of interest.

## Data Availability

The data used to support the findings of this study were obtained from the data of patients with lung squamous cell carcinoma in the TCGA database (https://portal.gdc.cancer.gov/). It is an open data resource.
